# Different Clinical Manifestations of Adenoviral Infection Confirmed Using Point-of-Care Testing in a Group of Hospitalized Children

**DOI:** 10.3390/pediatric15010001

**Published:** 2022-12-22

**Authors:** Katarzyna Ptak, Izabela Szymońska, Anna Olchawa-Czech, Zuzanna Baliga, Marta Sawina, Agata Woźnica, Przemko Kwinta

**Affiliations:** 1 Department of Pediatrics, Jagiellonian University Medical College, 30-663 Cracow, Poland; 2 Faculty of Medicine, Jagiellonian University Medical College, 30-663 Cracow, Poland

**Keywords:** adenovirus, fever, children, point-of-care test, infection

## Abstract

Objective: A restrospective analysis of the clinical picture (inflammatory markers, characteristics of fever, comorbidities) in different clinical manifestations of human adenovirus (HAdV) infections confirmed using point-of-care testing in a group of hospitalized children. Material and Methods: A total of 135 children with confirmed HAdV infections were divided into three groups according to their clinical symptoms: Group A—respiratory (*n* = 57), Group B—gastrointestinal (*n* = 40), and Griup C—mixed (*n* = 38). Results: Respiratory and mixed HAdV-infected patients, as compared with gastrointestinal HAdV-infected patients, were younger (median value (Me) and interquartile range (IQR) (months): 17 (12–30) and 17 (12–27) vs. 30 (16–50), *p* = 0.04), had a longer duration of fever (days): 3 (1–5) and 3 (1–4) vs. 1 (1–2), *p* = 0.01), and had higher C-reactive protein values (mg/L): 29.2 (10.4–69.1) and 28.7 (10.8–49.1) vs. <5 (<5–20.6), *p* < 0.001). There were no correlations between CRP levels and patient’s age, fever duration, the occurrence of acute otitis media and lower respiratory tract infection, and antibiotic treatment before admission. Conclusions: Patients with respiratory HAdV infections have fevers more often, the duration of the fever prior to admission is longer, and CRP levels are higher.

## 1. Introduction

Human adenoviruses (HAdVs) are important causes of infections in young children. The incidence ranges from 7 to 10% in pediatric patients with respiratory tract infections, but the precise rate is unknown because diagnostic testing is not commonly performed [1,2,3]. HAdV infections are associated with a wide range of respiratory and/or gastrointestinal (GI) tract symptoms and can cause fatal infections in healthy and immunocompromised patients [1].

HAdVs are double-stranded DNA viruses, divided into more than 55 distinct serotypes and other “new” genetic variants, classified into seven species (A–G). Some types are associated primarily with respiratory tract diseases (1–5, 7, 14, and 21) whereas others with gastroenteritis (40 and 41) [1,4]. In clinical practice, mixed symptoms are often seen. HAdVs can be transmitted via respiratory tract secretions, fecal-oral spread, and by exposure to infected tissue or blood. Children with respiratory or generalized infections excrete the virus for 3 to 6 weeks in throat mucosa or stool [1,5]. Consequently, HAdVs spread easily among children and people living in high-density areas and can be a serious threat to patients with immunodeficiencies [5]. 

Symptomatic HAdV infections are commonly characterized by high-grade and long-lasting fevers with elevated inflammatory cytokines [1,6]. As a result, a high C-reactive protein (CRP) level may be noted which can often lead to unnecessary use of antibiotics and hospital admissions. Using of point-of-care (POC) testing for HAdV infections could improve the pre-hospital evaluation, provide the diagnosis, and lead to appropriate therapeutic decisions [7,8]. 

This study aimed to define the clinical picture (inflammation markers, characteristics of fever, and comorbidities) in the different clinical manifestations of HAdV infections confirmed using serologic POC testing in a group of hospitalized children.

## 2. Material and Methods

### 2.1. Study Design

A retrospective, single-center study conducted at the Department of Pediatrics, Jagiellonian University Medical College, Cracow—a pediatric tertiary care center. 

### 2.2. Patient Identification and Selection

The electronic database was searched for patients between the ages of 0 and 18 years diagnosed with HAdV infections who had been admitted to the Department of Pediatrics between 1 February 2016 and 1 November 2020. For each patient, a defined set of data (age, CRP level, white blood count, symptoms, and length of hospitalization) was extracted from the hospital medical record. Patients were excluded if at least one of the following criteria were met: co-infection with any other diagnosed respiratory or GI virus, urinary tract infection, a positive blood culture, or any positive culture from an otherwise normal sterile site.

HAdV infections were confirmed on the first day of admission based on a positive result indicating the presence of HAdV antigen from a nasopharyngeal swab or sample of stool using the following tests:

1. Nasopharyngeal swab: *Certest Biotec S.L., Spain, Influenza A+ B + RSV + Adeno.* (detects serotypes 2, 3, 4, 5, 15, 31, 40, 41). The specimens were collected by trained medical doctors. Results were measured by POC tests.
2. Stool: *Certest Biotec S.L., Spain, Influenza A+ B + RSV + Adeno.* (detects serotypes 2, 5, 15, 31, 40, 41). The test was performed in the microbiology lab of the University Children’s Hospital of Cracow.

### 2.3. Definitions of the Groups of Patients

Based on the clinical presentation of HAdV infections, the following groups were defined:

Group A: Patients with only respiratory tract symptoms including acute otitis media (AOM), upper respiratory tract infections (e.g., conjunctivitis, rhinitis, pharyngitis, tonsillitis, and laryngitis), and lower respiratory tract infections (LRTI, eg. bronchitis, bronchiolitis, and pneumonia). The diagnosis of LRTI was made clinically and/or by using an imaging method (such asw USG or chest-X ray).

Group B: Patients with only GI symptoms (including diarrhea and vomiting).

Group C: Patients with both respiratory and GI symptoms.

### 2.4. Statistical Methods

The Shapiro–Wilk test was used to test for the normal distribution of continuous variables. Because this test indicated that the null hypothesis (distribution is normal) should be rejected in most of the analyzed variables, the Kruskal–Wallis test was used. The chi-square test was used for the comparison of categorical variables. A *p*-value of <0.05 was considered to be statistically significant. The statistical analysis was performed using the IBM SPSS Statistics v. 27 software (Armonk, NY, USA).

## 3. Results

A total of 135 children with HAdV infections were identified. Among them, 57 (42.2%) patients presented with only respiratory symptoms, 40 (29.6%) patients presented with only GI symptoms, and 38 (28.1%) patients had both respiratory and GI symptoms. None of our patients had severe HAdV infections (including intensive care admission, hepatitis, myocarditis, need for mechanical ventilation, or death). A comparison of selected demographic and clinical variables among the studied groups is presented in Table 1.

There were several differences between groups A and C as compared with group B. Children from groups A and C were younger and more feverish (incidence of and duration of fever) before admission. Additionally, these patients were more often treated with antibiotics prior to admission. There was no difference in the length of hospitalization among the groups (*p* = 0.14). 

In group A, nasopharyngeal swabs were positive in 82% of patients, and in group B stool samples were positive for HAdV antigen in 82.5% of patients. An interesting observation was that in 17.5% of children in group A, the HAdV etiology was recognized based on the stool sample test. Likewise, in 20% of children from group B, the HAdV etiology was recognized based on a nasopharynx swab. These results are presented in Table 2.

### Laboratory Results

A comparison of selected laboratory variables among studied groups is presented in Table 3. The relationships between CRP levels and fever, LRTI, AOM, and antibiotic therapy were analyzed and are presented in Table 4.

CRP levels tended to be lower in group B regardless of age, but due to limited sample size, the evidence for population-level differences was not conclusive in the youngest age category. In the older group of patients, respiratory symptoms were associated with high CRP levels.

There was a difference in observed absolute lymphocyte counts (ALCs) among children from group B who had significantly lower ALC values (*p* = 0.001). There was no difference in white blood cell and absolute neutrophil values.

Patients with fever before admission had higher CRP levels. Children who received antibiotics prior to admission, as compared with children who had not, had similar CRP levels in groups A and C but higher CRP levels in group B. There was no difference in observed CRP levels among children who received antibiotics prior to admission among groups A, B, and C. Children with LRTI and AOM had similar CRP levels in groups A and C. Respiratory symptoms (groups A and C), even without a diagnosed LRTI, and AOM were associated with higher CRP levels as compared with group B.

Finally, the prevalence of an abnormal CRP result within the groups was analyzed. A summary of the analysis is presented in Table 5. There was a difference in observed CRP levels in children with respiratory symptoms who more often had CRP levels >15 mg/dL (*p* = 0.002) and >100 mg/dL (*p* = 0.016). Moreover, in groups A and C, 68% of patients had a CRP level >15 mg/dL and 21% of patients in group A had a CRP level >100 mg/dL. 

## 4. Discussion

HAdV infections are one of the most common causes of fever requiring hospitalization when it occurs in children under the age of five [4,7,9]. The course of infection is characterized by a broad spectrum of symptoms from mild to persistent, including multi-day fevers and threatening infections. An early and accurate diagnosis of a viral infection allows the avoidance of unnecessary antibiotic therapy and calms down the doctor, parents, and the patient. Using POC testing for HAdV infections is readily available and allows doctors to make the correct diagnosis quickly. However, POC tests are still not performed routinely [3,10,11]. 

In our study, we analyzed the clinical picture of HAinfections in the context of the most frequently ordered outpatient blood tests including CRP and CBC, and the clinical manifestations of the infection.

In our study, 91.9% of patients were children under the age of five. This is consistent with other authors’ observations. In the study carried out by Shachor-Mayouhas Y and co-authors [12], a group of immunocompetent hospitalized patients, including those in the intensive care unit, due to respiratory system infections caused by the HAdV found that 90% of the children were under five years of age (median 1.16 years old). 

Similarly, in case-control studies carried out by Fan-Zhou and co-authors [13], a group of children hospitalized due to gastroenteritis caused by the HAdV found that 91.14% of patients were under three years of age. Our patients with respiratory tract symptoms were substantively significantly younger than patients with symptoms concerning the digestive tract. These patients also had more often and longer periods of fever. As a result, a young child with high-grade fevers for multiple days and respiratory tract symptoms provides doctors with a picture of a serious infection and strong suspicion of bacterial etiology [14]. Greater antibiotic use among patients in this group prior to hospital admission confirms this assumption.

What is more, patients presenting with symptoms of a respiratory system infection as compared with patients presenting with symptoms concerning a digestive system infection have higher CRP levels including substantively more CRP levels above 100 mg/dL. This additionally hinders decision-making in clinical practice. The CRP level traditionally indicates a bacterial infection. Without another etiology explaining the clinical picture, it may result in the initiation of antibiotic therapy. 

In most patients (82.4%) with respiratory tract symptoms, HAdV infections were confirmed by nasopharyngeal swabs, whereas in patients with digestive tract symptoms a stool sample test confirmed the diagnosis (82.5%). These tests are slightly different in terms of detecting serotypes 3 and 4 by using a nasopharyngeal swab. Serotypes 3 and 4 are believed to primarily cause HAdV infections with respiratory symptom manifestations. After analyzing available sources, studies concerning the relationships among isolated 3 and 4 HAdV serotypes, high laboratory values, and severe infections were not found. Infections caused by additional serotypes detected by nasopharyngeal swabs may not be related to higher indicators of inflammation. However, HAdV serotype infections with respiratory symptoms could cause a higher increase in CRP concentration. This is consistent with observations in a study carried out by Mistchenko and co-authors [15], in which higher IL-6 and TNF-alfa concentrations were found in patients with severe respiratory tract infections caused by HAdVs. Similarly, in research by Kawasaki and co-authors [16], significantly higher CRP and IL-6 concentrations were found in patients with respiratory system infections caused by HAdVs as compared with infections caused by influenza viruses or respiratory syncytial viruses (RSV). We analyzed bacterial superinfection as a cause of the higher CRP levels. In our study, such a relationship was not found. Statistical differences in CRP levels among patients receiving antibiotics prior to hospital admission as compared with those not receiving antibiotic was not found. Therefore, it can be assumed that the decision whether to put a patient on antibiotics was not made due to higher CRP levels but due to the child’s condition. 

Sharing the knowledge about high CRP levels in HAdV infections, routinely using the clinical scale to assess the risk of streptococcal pharyngitis infections (Centro-McIzaak scale), as well as using POC testing more broadly (e.g. Strep-test, antigenic tests for RSV, and adenovirus, influenza) to confirm bacterial or viral infections may allow for rational antibiotic therapy use [14]. 

In the presented study, HAdV infections causing digestive tract symptoms were not associated with significant increases in CRP levels. This may suggest that serotypes connected with digestive tract infections employ different penetration mechanisms to get inside a cell [17]. Inflammatory response activation mechanisms in infections caused by HAdVs may depend on the tissue that has been infected; however, this requires further study. 

The fact that in two cases, infections were confirmed on both tests is an interesting observation. It may be due to technical errors or different viral antigen releases from different systems depending on the time of infection. It also confirms the observation that the course of viral infections in small children is often accompanied by nonspecific symptoms from different organs regardless of the primary tropism of the HAdV serotype. In a case-control study carried out by Fang-zhou Qiu and co-authors [13], HAdV3 genetic material was found in stool samples of patients who presented with diarrhea, which is classically believed to cause respiratory system infections.

Our study has significant limitations. It was a retrospective study. The diagnosis was made by antigen tests. On the one hand, the gold standard for diagnosing viral infections should be a polymerase chain reaction (PCR) test, which is more sensitive and allows for the identification of the particular HAdV serotype [18]. The use of PCR tests, however, is limited by their high price (especially multi-panel tests) and limited accessibility [18]. On the other hand, a rapid, positive result on antigen POC tests and the awareness that HAdV infections can cause high inflammatory markers can enable quick clinical decisions [8,10]. The HAdV infections were only checked on the first day of admission. Repetitive testing of patients with clinical signs suggested adenoviral infection could increase the number of positive diagnoses.

## 5. Conclusions

Patients with HAdV infections and respiratory symptoms as compared with patients with GI symptoms are more feverish (more frequent and longer fevers prior to admission) and have higher CRP levels. In the group of children with respiratory presentations, there was no correlation between CRP level and duration of fever, the occurrence of AOM, LRTI symptoms, and the age of the patient. Antibiotic treatment prior to admission was very common in the group of patients with respiratory symptoms. Early use of POC tests to detect the most common viral antigens as well as sharing the knowledge about the natural course of HAdV infections (respiratory system infections are accompanied most commonly by high infection markers) may have an impact on the rational application of antibiotic therapy. 

## Figures and Tables

**Table 1:** Demographic and clinical characteristics of patients with adenoviral infection.

	Group A (*n* = 57)	Group B (*n* = 40)	Group C (*n* = 38)	*p*
Age (months; Median; IQR)	17 (12–30)	30 (16–50)	17 (12–27)	0.04 *
Age <12 months 12–24 months >24 months	15 23 19	4 14 22	9 19 10	0.066 #
Fever before admission n (%)	54 (94.7%)	16 (40%)	30 (78.9%)	<0.001 #
Antibiotics before admission n (%)	24 (42.1%)	3 (7.5%)	14 (36.8%)	0.001 #
Duration of fever before admission only feverish patients (days; median; IQR)	3 (1–5)	1 (1–2)	3 (1–4)	0.01 *
LOS (days), median (IQR)	4 (3–6)	3 (3–5)	4 (3–5)	0.14 *

* *p*-value for Kruskal–Wallis test; # *p*-value for chi-square test.

**Table 2:** Type of adenoviral tests used to confirm the diagnosis in groups.

	Group A (*n* = 57)	Group B (*n* = 40)	Group C (*n* = 38)	*p* *
Positive nasopharyngeal swab for adenovirus antigen	47	7	22	<0.01
Positive stool test for adenovirus antigen	10	32	15	<0.01
Positive both tests for adenovirus antigen	0	1	1	<0.01

* *p*-value for chi-square test.

**Table 3:** The characteristics of laboratory values in patient groups.

	Group A (*n* = 57)	Group B (*n* = 40)	Group C (*n* = 38)	*p* *
WBC (×10^3^/μL Median; IQR)	13.5 (10.3–18.9)	12.3 (8.1–15.2)	13.7 (9.2–18.7)	0.29
ANC (×10^3^/μL Median; IQR)	6.8 (5–10.9)	6.8 (4.7–10.7)	6.4 (4.4–12.7)	0.98
ALC (×10^3^/μL Median; IQR)	4.5 (2.9–6.6)	2.8 (1.6–4.1)	4.3 (2.9–5.9)	0.001
PLT (×10^3^/μL Median; IQR)	308 (251–370)	332 (273–405)	325 (289–363)	0.36
CRP (mg/L; Median; IQR)	29.2 (10.4–69.1)	<5 (<5–20.6)	28.7 (10.8–49.1)	<0.001
CRP among age groups (mg/L; Median; IQR)				
<12 months	21.5 (5.4–28)	10.1 (<5–38.9)	29.2 (18.2–57)	0.566
12–24 months	37.4 (12.7–69)	<5 (<5–19.3)	23.5(7.2–46)	0.025
>24 months	30 (7–133)	<5 (<5–21.1)	31.3 (10.8–56.4)	0.013

* *p*-value for Kruskal–Wallis test; WBC—white blood count; ANC—absolute neutrophil count; ALC—absolute lymphocyte count; PLT—plates.

**Table 4:** Clinical factor module CRP levels in studied groups.

	Group A (*n* = 57)	Group B (*n* = 40)	Group C (*n* = 38)	*p* *
Children with fever children (mg/L; median; IQR)	30.2 (10.4–69.2)	19.9 (13.3–6.3)	40.6 (22–57)	0.721
Children without fever (mg/L, median; IQR)	15.7 (5.2–21.1)	<5 (<5–<5)	<5 (<5–10)	0.019
Children who received antibiotics prior to admission (mg/L; median; IQR)	29 (11.6–59)	34 (<5–64)	35.3 (14–49)	0.97
Children without antibiotics prior to admission (mg/L; median;IQR)	29.2 (6.9–121)	<5 (<5–19.3)	26.4 (9.8–56)	0.001
Children with LRTI (mg/L; median; IQR)	24.3 (9–46)	N/D	28.2 (9.5–49.1)	1.0
Children without LRTI (mg/L;median;IQR)	30.2 (10.4–115.6)	<5 (<5–20.6)	35.8 (22.7–57.5)	0.001
Children with AOM (mg/L; median; IQR)	69.1 (12.8–154)	N/D	24.9 (5–44.8)	0.32
Children without AOM (mg/L;median;IQR)	27.7 (9–60)	<5 (<5–20.6)	28.7 (12–52.6)	0.001

* *p*-value for Kruskal–Wallis test; LTRI—lower respiratory tract infection; AOM—acute otitis media; N/D—no data.

**Table 5:** The characteristics of CRP levels among groups.

	Group A (*n* = 57)	Group B (*n* = 40)	Group C (*n* = 38)	*p* *
CRP > 15 mg/L	39 (68%)	14 (35%)	26 (68%)	0.002
CRP > 100 Mg/L	12 (21%)	1 (2.5%)	3 (7.8%)	0.016
CRP > 15 Mg/L				
<12 months	10 (66%)	2 (50%)	7 (77%)	0.6
12–24 months	16 (70%)	5 (36%)	12 (63%)	0.11
>24 months	13 (65%)	7 (32%)	7 (70%)	0.044
CRP > 15 Mg/L				
Fever	37 (67%)	12 (75%)	24 (80%)	0.44
No fever	2 (67%)	2 (8%)	1 (14%)	0.08

Data are presented as *n*/%; * *p*-value for chi-square test.

## Data Availability

Not applicable.
